# Structured Clinical Supervision in Perioperative Nursing: A Systematic Review of Its Impact on Professional Development and Patient Safety

**DOI:** 10.3390/nursrep16060197

**Published:** 2026-06-08

**Authors:** Marisa de Paula, Diogo Folgado, Ana João, Ana Madeira

**Affiliations:** 1 Unidade Local de Saúde da Lezíria, 2005-177 Santarém, Portugal; 2 Red Cross Hospital, 1549-008 Lisboa, Portugal; diogo_folgado@hotmail.com; 3 Escola Superior de Saúde de Santarém, Instituto Politécnico de Santarém, 2001-904 Santarém, Portugal; ana.joao@essaude.ipsantarem.pt (A.J.); ana.madeira@essaude.ipsantarem.pt (A.M.); 4 Comprehensive Health Research Center (CHRC), Universidade de Évora, 7000-811 Évora, Portugal; 5 RISE-Health, School of Health, Santarem Polytechnic University, 2005-075 Santarem, Portugal

**Keywords:** clinical supervision, perioperative nursing, professional development, patient safety, quality of care

## Abstract

**Background**: The perioperative context is characterized by high complexity and a significant risk of adverse events, requiring highly developed technical and non-technical competencies. Structured clinical supervision has been identified as a relevant strategy for professional development and for promoting the quality and safety of care, although the specific evidence in this context remains dispersed. **Objective**: To analyze the available scientific evidence on the impact of structured clinical supervision on nurses’ professional development and on the quality and safety of care delivered in the perioperative setting. **Methods**: A systematic literature review was conducted in accordance with PRISMA 2020 recommendations. The search was performed in the PubMed, Web of Science, EBSCO, SciELO, BVS, and CONSENSUS databases and included studies published between January 2020 and October 2025 in Portuguese, English, or Spanish with full-text availability. The research question was structured according to the PICO strategy. Study selection was carried out in multiple stages (duplicate removal, screening by title and abstract, and full-text review), performed by two independent reviewers. Methodological quality was assessed using the Joanna Briggs Institute (JBI) checklists. Data synthesis was conducted through thematic narrative analysis, given the methodological heterogeneity of the included studies. **Results**: Twelve studies were included, predominantly qualitative and observational in nature, as well as psychometric validation studies, one Delphi study, and one quasi-experimental study. The findings show consistent convergence regarding the association between structured clinical supervision and the development of technical and non-technical competencies, namely communication, leadership, teamwork, situational awareness, and decision-making. The use of structured assessment instruments demonstrated good psychometric reliability and improved the quality of supervisory feedback. Organizational factors, such as protected time, specific training for supervisors, and role clarification, were identified as determinants of the effectiveness of the supervisory process. However, the predominance of non-experimental designs and the scarcity of objective clinical outcomes limit direct causal inference between structured supervision and measurable reduction in adverse events. **Conclusions**: The available evidence suggests that structured clinical supervision is a relevant component for the professional development of perioperative nurses and for strengthening the safety culture in the operating room. Despite the high conceptual consistency of the findings, the overall strength of evidence is moderate, and experimental and longitudinal studies are needed to consolidate the impact of supervision on objective clinical indicators of care quality and safety.

## 1. Introduction

Patient safety in the perioperative setting remains a major challenge due to the technical complexity of surgical care, the vulnerability of patients, and the need for rapid and coordinated decision-making. The operating room is a high-risk environment characterized by invasive procedures, advanced technologies, multidisciplinary interaction, and time-sensitive clinical demands, requiring perioperative nurses to demonstrate highly developed technical and non-technical competencies [1]. In this context, nurses play a critical role in infection prevention, medication safety, interprofessional communication, coordination of surgical activities, and management of emergency situations, all of which are essential for reducing adverse events and ensuring safe care [2].

Despite advances in surgical safety practices, perioperative incidents continue to represent a substantial proportion of hospital adverse events. Persistent failures were identified across the perioperative pathway, particularly during transitions of care, where communication and coordination deficits remain significant contributors to patient harm [3]. Similarly, the organizational complexity and fragmentation of perioperative processes increase the risk of errors and compromise patient safety [4]. These findings reinforce the importance of structured safety systems and consistent professional support mechanisms within perioperative practice.

Within this framework, clinical supervision has emerged as an important organizational and educational strategy to support professional performance and safe clinical practice. Supervision is seen not only as a mechanism for monitoring clinical activities but also as a structured process that promotes guidance, reflective practice, and professional development [5]. Evidence suggests that structured supervision and well-established safety systems positively influence perioperative nursing safety activities, adherence to protocols, interprofessional communication, and the early identification of potential adverse events [5,6,7]. Furthermore, nurses’ understanding of their responsibilities within the operating room is closely associated with the adoption of safety practices, highlighting the importance of clear supervisory processes [8].

Clinical supervision is a multidimensional construct widely conceptualized in nursing as a formal, structured, and reflective process through which experienced professionals support the development of clinical competence, professional judgment, reflective practice, and patient safety [9,10]. According to Proctor’s framework, clinical supervision encompasses three interrelated functions: normative (quality and accountability), formative (learning and competence development), and restorative (professional support and wellbeing) [9]. This model remains one of the principal theoretical frameworks underpinning contemporary approaches to supervision in nursing practice.

In perioperative settings, which involve technical complexity, rapid decision-making, and high-risk situations, structured clinical supervision may therefore operate as an integrative mechanism linking education, reflective practice, professional accountability, and safe clinical performance [5]. The supervisory process contributes not only to competency development but also to the reinforcement of communication, situational awareness, teamwork, and adherence to safety standards within complex surgical environments.

However, clinical supervision should be distinguished from related educational concepts frequently used in perioperative nursing literature, particularly preceptorship and mentorship [11]. Preceptorship generally refers to a time-limited, practice-oriented relationship in which an experienced clinician supports the transition and clinical integration of a student or newly qualified nurse, with emphasis on role socialization, acquisition of practical competencies, and adaptation to the clinical environment [12,13]. Mentorship, in contrast, usually involves a broader and longer-term developmental relationship centered on career progression, professional identity formation, psychosocial support, and professional empowerment. Clinical supervision differs from both concepts by emphasizing structured reflection on practice, systematic feedback, critical thinking, and ongoing professional development within clinical governance and patient safety frameworks [9].

For the purposes of this review, structured clinical supervision was operationally defined as formalized supervisory processes characterized by explicit educational objectives, planned feedback, reflective practice, supervisor involvement, and/or the use of structured assessment or supervisory frameworks. Educational technologies such as simulation-based learning, immersive virtual reality, and structured assessment instruments were not considered supervision per se but rather complementary pedagogical strategies capable of strengthening, supporting, or operationalizing supervisory processes in perioperative settings [14,15].

Despite its recognized relevance, supervision practices in the operating room remain frequently informal, heterogeneous, and poorly structured, which limits their effectiveness and hinders the consolidation of a systematic safety culture [9]. There is also a scarcity of studies that explore in depth the specific impact of structured clinical supervision in the perioperative environment. Although recent research addresses safety practices and care guidelines [3,4], few studies examine how supervisory processes influence the implementation of these recommendations or the development of the professional competencies required to operate in a high-acuity context. Studies on nursing supervision in general hospital settings [16] point to relevant organizational effects but do not specifically address the operating room, which presents distinct technical, relational, and organizational characteristics.

Further exploration of this topic is therefore essential, particularly in light of the growing complexity of perioperative care and the persistent gap between educational interventions and demonstrable patient safety outcomes. Although structured clinical supervision is consistently associated with improvements in nurses’ technical and non-technical competencies, the current body of evidence remains methodologically heterogeneous and largely dominated by qualitative and observational designs, limiting the strength of causal inference and the translation of findings into measurable clinical impact. This disconnect highlights the need for a rigorous synthesis capable of critically integrating existing knowledge and clarifying its implications for practice.

Literature reviews play an essential role in consolidating scientific knowledge by identifying the current state of evidence, critically appraising methodological limitations, and guiding future research priorities. Literature reviews contribute to the definition of research problems, the identification of knowledge gaps, and the advancement of scientific understanding within a given field [17]. In particular, systematic reviews provide a structured and transparent method for synthesizing evidence derived from diverse methodological approaches, enabling a more comprehensive understanding of complex phenomena such as clinical supervision in nursing practice.

Although perioperative education, patient safety, non-technical skills, and supervisory practices have been explored in previous studies, no recent systematic review has specifically examined the impact of structured clinical supervision on perioperative nursing practice and patient safety outcomes. Existing literature has primarily focused on isolated educational interventions, simulation-based learning, preceptorship experiences, or assessment instruments, often without clearly distinguishing these approaches from structured clinical supervision itself. Consequently, important gaps remain in understanding how supervisory processes, organizational factors, structured assessment frameworks, and complementary educational strategies operate within perioperative nursing practice. In particular, there is limited synthesis of evidence examining how structured clinical supervision contributes to the development of technical and non-technical competencies, reflective practice, supervisory consistency, and safety-related professional performance in operating room settings.

Therefore, considering the need to critically integrate the available evidence and address persistent limitations related to methodological rigor, outcome measurement, and contextual variability, the present systematic review is justified. By focusing specifically on the perioperative environment—a setting characterized by high clinical risk, organizational complexity, and demanding professional performance—this review seeks to bridge the gap between conceptual evidence and practical applicability.

The present systematic review aims to provide an integrative and conceptually structured synthesis of contemporary evidence regarding structured clinical supervision in perioperative nursing. Unlike previous literature centered on isolated educational or training interventions, this review specifically examines supervision as an organizational, pedagogical, and reflective process associated with professional development, competency enhancement, and patient safety within perioperative settings.

## 2. Materials and Methods

The reporting of this systematic review was conducted in accordance with the Preferred Reporting Items for Systematic Reviews and Meta-Analyses (PRISMA) 2020 guidelines. To ensure transparency, completeness, and methodological rigor, the PRISMA 2020 checklist was completed and is provided as Appendix A.

All relevant items of the checklist were addressed throughout the manuscript, including the structured presentation of the research question, eligibility criteria, search strategy, study selection process, data extraction, risk of bias assessment, and synthesis of results. Where specific items were not applicable—particularly those related to quantitative synthesis and meta-analysis—this was explicitly stated and justified, given the methodological heterogeneity of the included studies.

The PRISMA framework supported a systematic and reproducible approach to the identification, selection, appraisal, and synthesis of the available evidence, contributing to the robustness and clarity of the review process.

The review protocol was prospectively registered in the International Prospective Register of Systematic Reviews (PROSPERO)-Centre for Reviews and Dissemination, University of York, York, UK-under the registration number CRD420261354167. The protocol predefined the research question, eligibility criteria, search strategy, and methods for study selection, data extraction, and methodological quality appraisal, thereby minimizing the risk of bias. No major deviations from the registered protocol were identified during the conduct of the review.

The review process began with the formulation of a structured research question based on the PICO framework, which guided the definition of the population, intervention, comparison, and outcomes.

Accordingly, the guiding research question was defined as follows: “In operating room nurses, does the implementation of a structured clinical supervision model contribute to professional development and to the improvement of care quality?”

The components of the research question were defined as follows:

P (Population): Operating room nurses;

I (Intervention): Implementation of a structured clinical supervision model;

C (Comparison): Absence of structured supervision or informal practices;

O (Outcomes): Professional development and improvement in the quality and safety of care.

The objective was to analyze the impact of structured clinical supervision on nurses’ professional development and on the quality and safety of care delivered in the perioperative setting.

### 2.1. Search Strategy

The systematic literature search was conducted between 17–20 October 2025, across the following electronic databases: PubMed (National Library of Medicine, Bethesda, MD, USA), Web of Science (Clarivate Analytics, Philadelphia, PA, USA), EBSCOhost-Information Services, Ipswich, MA, USA (CINAHL and MEDLINE Complete), SciELO, BVS, and CONSENSUS (Consensus AI, Boston, MA, USA; available at: https://consensus.app; accessed on 20 October 2025).

A comprehensive search strategy was developed a priori and adapted to the specific indexing systems, controlled vocabularies, and search functionalities of each database in order to ensure appropriate sensitivity and specificity. The search strategy combined MeSH descriptors, free-text terms, and Boolean operators related to perioperative nursing, clinical supervision, preceptorship, professional development, nursing education, and patient safety.

The following descriptors and keywords were used in different combinations according to database-specific requirements: Nurse, Nursing, Perioperative Nursing, Operating Room Nursing, operating room, operating theater, surgery, clinical supervision, preceptorship, Continuing Nursing Education, Nursing Education, Quality of Health Care, Staff Development, professional development, pedagogical supervision in nursing, clinical-based training, and clinical placement supervision.

The base search expression was structured as follows:

(Nurs* OR “Perioperative Nursing” OR “Operating Room Nursing”) AND (“operating room” OR “operating theatre” OR surgery) AND (preceptorship OR “clinical supervision”) AND (“Continuing Nursing Education” OR “Nursing Education” OR “Quality of Health Care”).

The search strategy included both “clinical supervision” and “preceptorship”-related terminology in order to maximize search sensitivity and capture supervisory practices described using different terminologies within perioperative nursing literature. However, conceptual distinctions between preceptorship, mentorship, and clinical supervision were maintained throughout the data extraction and interpretive synthesis processes. Consequently, only studies addressing structured supervisory processes involving reflective practice, feedback, competency development, or formalized supervisory frameworks were interpreted within the conceptual domain of structured clinical supervision.

In addition to traditional indexed bibliographic databases, the CONSENSUS platform was consulted as a complementary search resource to enhance the sensitivity of the search process and identify potentially relevant studies not readily retrieved through conventional database indexing. CONSENSUS is an artificial intelligence-assisted academic search tool that aggregates scientific literature from multiple scholarly sources and supports thematic exploration through semantic search functionalities. Its use in the present review was intended exclusively as a supplementary strategy for literature identification and not as a substitute for indexed database searching. All records identified through CONSENSUS were subjected to the same screening procedures, eligibility criteria, duplicate removal process, and critical appraisal applied to studies retrieved from the primary databases.

To ensure methodological transparency and reproducibility, the complete search strategies used for each database, including Boolean operators, filters, limits, execution dates, and number of records retrieved, are provided in Appendix A (Table A1).

### 2.2. Inclusion and Exclusion Criteria

The inclusion criteria comprised primary studies published between January 2020 and October 2025, available in Portuguese, English, or Spanish, with full-text access, that focused on clinical supervision, supervisory practices, or professional development in nursing within perioperative or operating room settings. Studies that did not address the research question, lacked thematic relevance, or were identified as non-scientific literature following full-text assessment were excluded.

The temporal delimitation of the review was established a priori during the methodological protocol design phase. Although clinical supervision in nursing is a well-established field of research, the present review specifically focused on contemporary structured approaches to supervision within perioperative environments, particularly those integrating formal assessment frameworks for non-technical skills, simulation-based educational methodologies, reflective practice, and supervisory models aligned with current patient safety and quality-of-care standards.

The decision to include studies published from 2020 onward was supported by the substantial evolution observed in perioperative education and supervisory practices in recent years. This period has been characterized by increased scientific production related to supervision in high-complexity clinical settings, driven by organizational, technological, and pedagogical transformations, as well as by the systemic impact of the COVID-19 pandemic on clinical learning processes and supervisory dynamics. Recent literature has also demonstrated growing emphasis on structured competency assessment, interprofessional collaboration, reflective learning, and the integration of patient safety principles into perioperative nursing supervision.

The adoption of a contemporary time frame aimed to ensure the synthesis of evidence consistent with current clinical practices, organizational realities, and educational models while reducing conceptual heterogeneity associated with historical or less structured forms of supervision that differ substantially from current implementations.

Only studies with full-text availability were included to enable comprehensive methodological appraisal, rigorous data extraction, and consistent assessment of study quality. It is acknowledged that this criterion may have introduced potential selection bias by excluding potentially relevant studies not accessible in full text. Nevertheless, full-text availability was considered essential to ensure methodological rigor, reliability, and interpretive consistency in the synthesis of evidence.

Additionally, it is recognized that restricting the review to recent publications may have excluded earlier theoretical and empirical contributions relevant to the historical development of clinical supervision in nursing. However, considering the analytical focus on contemporary structured supervisory models and their applicability to current perioperative practice, this strategy was considered appropriate to enhance the practical relevance, methodological coherence, and timeliness of the synthesized findings.

### 2.3. Study Selection Process

**Figure 1:**
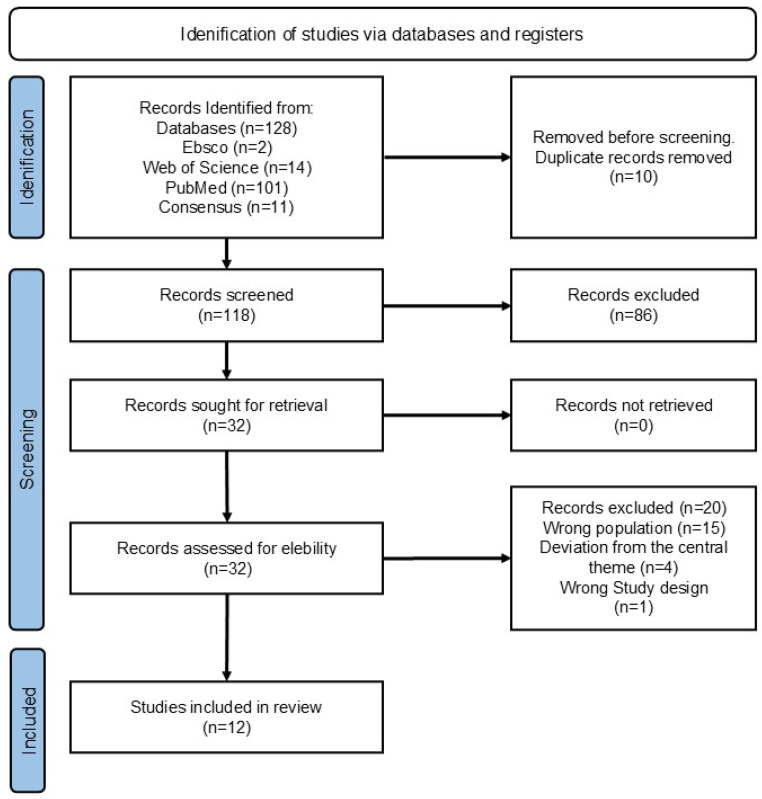
PRISMA 2020 Diagram.

Study selection was carried out in sequential stages: duplicate removal, title screening, abstract screening, and full-text assessment. The identification and selection process is presented in the PRISMA 2020 flow diagram, ensuring traceability from the number of records identified to the final number of included studies. Screening (title, abstract, and full text) was performed independently by two reviewers. Disagreements were resolved through discussion and consensus; when consensus could not be reached, a third reviewer was consulted (Figure 1). The duplication and screening process was supported by the Rayyan platform (Qatar Computing Research Institute, Doha, Qatar; available at https://www.rayyan.ai; accessed on 20 October 2025).

Following the full-text review of the eligible articles, it was identified that one of the included documents corresponded to a professional report/opinion paper. Given its relevance for understanding supervision practices in the perioperative context, this document was retained as contextual support and analyzed separately from the empirical evidence.

### 2.4. Methodological Quality Assessment and Risk of Bias

Methodological quality and risk of bias were independently assessed using the Joanna Briggs Institute (JBI) Critical Appraisal Tools (2024 version). Given the methodological heterogeneity of the included studies, appraisal instruments were selected according to the specific design of each study to ensure rigorous and design-appropriate evaluation.

The appraisal process was conducted independently by two reviewers using the JBI checklists corresponding to each methodological design. The following instruments were applied:

- JBI Checklist for Analytical Cross-Sectional Studies (cross-sectional, correlational, psychometric validation, and secondary data analysis studies);
- JBI Checklist for Quasi-Experimental Studies (pre–post intervention studies);
- JBI Checklist for Qualitative Research (qualitative interview-based studies);
- JBI Checklist for Text and Opinion Papers (professional reports and expert opinion documents).

Each study was evaluated criterion by criterion, with items classified as “Yes”, “No”, “Unclear”, or “Not Applicable”, in accordance with JBI recommendations. Methodological quality was subsequently calculated based on the proportion of applicable criteria fulfilled.

To facilitate interpretive synthesis across heterogeneous methodologies, studies were descriptively grouped according to predefined quality thresholds. Although these categories are not part of the official JBI nomenclature, they were operationally adopted to support comparative interpretation of the evidence. Studies were classified as follows:

- High quality (≥80% of criteria met);
- Moderate–high quality (70–79%);
- Moderate quality (60–69%);
- Moderate–low quality (50–59%);
- Low quality (<50%).

All assessments were performed independently by two reviewers. Discrepancies were discussed until consensus was achieved, and when necessary, a third reviewer was consulted to resolve disagreements. Although inter-rater agreement coefficients were not calculated, consensus procedures were systematically applied throughout the appraisal process to enhance methodological consistency and reliability.

No studies were excluded solely based on methodological quality. However, the limitations identified during critical appraisal were explicitly considered during data synthesis and interpretation of findings. Table 1 summarizes the methodological appraisal, including the number of criteria fulfilled and the corresponding descriptive quality classification assigned to each study.

As shown in Table 1, the overall body of evidence was predominantly characterized by moderate to moderate–high methodological quality, including one Delphi study classified as high quality and one cross-sectional study classified as low quality.

- Several methodological strengths were identified across the included studies:
- Clearly defined research objectives aligned with the methodological design;
- Use of appropriate statistical analyses in quantitative studies;
- Robust psychometric validation procedures in methodological studies;
- Adequate representation of participants’ perspectives in qualitative studies;
- Ethical approval and informed consent reported in empirical studies.

The most frequent methodological limitations included:

1. Selection bias, particularly in studies using convenience sampling or voluntary participation;
2. Measurement bias, when non-validated instruments or tools specifically developed for the study were used;
3. Confounding bias, as most observational studies did not perform multivariable adjustment;
4. Limitations in causal inference, particularly in quasi-experimental studies without a control group;
5. Limitations in reflexivity, as qualitative studies did not explicitly address the researcher’s positioning.

The study classified as low quality presented significant methodological limitations, namely the absence of clearly defined inclusion criteria and the use of non-validated instruments. Its findings were therefore interpreted with caution in the final synthesis.

Although the professional report fully met the JBI checklist criteria for Text and Opinion, it does not constitute empirical evidence and was interpreted as a contextual contribution grounded in practice.

### 2.5. Data Assessment and Analysis

Data extraction and analysis involved full-text reading of the eligible studies and the systematic extraction of relevant information to address the research question. Data were collected on the characteristics of the included studies, namely authors, year of publication, country of origin, methodological design, participants, clinical context, instruments used, and main findings.

The data were synthesized through thematic narrative analysis, with the main conceptual domains emerging from the literature identified and organized. These included the development of technical and non-technical competencies, the training and role of supervisors, the use of structured supervision instruments, and the organizational conditions associated with supervisory practice.

The analysis process included:

- Data reduction, through the extraction and coding of essential information;
- Organization of data into comparative matrices, enabling the systematization of methodological characteristics and study findings;
- Comparison of included studies, aiming to identify patterns, convergences, and divergences among results;
- Data interpretation, allowing the integration of findings in a coherent manner consistent with the research question;
- Data synthesis was conducted in a descriptive and comparative manner, considering the methodological heterogeneity of the included studies, which precluded the performance of a meta-analysis.

The systematization presented below in Table 2 complements this analysis, providing a rigorous comparative overview that supports the interpretation of the results of the systematic review.

The full review protocol is available in the PROSPERO database, ensuring transparency and reproducibility of the methodological process.

### 2.6. Reporting Bias Assessment

Due to the methodological heterogeneity of the included studies and the absence of a meta-analysis, a formal assessment of reporting bias (e.g., publication bias) was not conducted. However, efforts were made to minimize potential bias through a comprehensive search strategy across multiple databases, the inclusion of studies in three languages (English, Portuguese, and Spanish), and the use of predefined eligibility criteria. Nevertheless, the possibility of publication bias cannot be excluded, particularly given the restriction to peer-reviewed articles and full-text availability.

## 3. Results

A total of 12 studies published between 2020 and 2025 met all eligibility criteria and addressed the research question. The body of evidence is characterized by marked methodological heterogeneity, encompassing exploratory qualitative studies, cross-sectional observational research, secondary data analyses, one Delphi consensus study, two psychometric validation studies, and one quasi-experimental study. The diversity of designs, populations, instruments, and clinical contexts precluded the conduct of a meta-analysis; therefore, a structured narrative synthesis was performed, integrating findings comparatively and critically appraising the consistency and strength of the evidence.

Geographically, most studies originated from Europe (66.7%), particularly Norway and Sweden, followed by contributions from North America (8.3%), Asia (8.3%), Africa (8.3%), and Oceania (8.3%). This distribution suggests that the conceptual development of perioperative clinical supervision has been primarily driven by healthcare systems with established traditions in educational research, patient safety, and formalized supervisory processes. However, the absence of studies from contexts with lower research density limits the global generalizability of the findings.

Across studies, there is a high level of conceptual convergence regarding the association between structured clinical supervision and the development of non-technical skills, particularly communication, leadership, teamwork, situational awareness, and decision-making. The thematic consistency across studies is robust, despite methodological differences. However, the overall strength of the evidence is predominantly moderate, reflecting structural limitations of the available empirical body, namely:

- Predominance of qualitative and cross-sectional observational studies;
- Scarcity of experimental or controlled quasi-experimental designs;
- Lack of longitudinal studies to assess the sustainability of effects;
- Limited use of objective clinical outcomes, including direct indicators of patient safety.

Thus, although there is clear conceptual coherence regarding the structuring role of clinical supervision in perioperative professional development, there remains insufficient causal evidence to robustly quantify the magnitude of its clinical impact.

The integrative analysis allowed the organization of findings into four interrelated core domains: (1) development of technical and non-technical competencies; (2) use of structured supervision and assessment instruments; (3) influence of organizational factors and supervisor qualification; and (4) pedagogical strategies complementary to clinical supervision. These domains do not operate in isolation but rather constitute a systemic model in which the effectiveness of supervision depends on the articulation between pedagogical, organizational, and evaluative components.

Overall, the findings indicate that the scientific field is at an advanced stage of conceptual consolidation but remains in methodological maturation. The available evidence supports the relevance of clinical supervision as an integrative axis between education, practice, and safety; however, it lacks studies with greater experimental rigor, multicenter samples, and objective measurement of clinical outcomes to consolidate its position within interventions with proven impact on perioperative safety.

### 3.1. Development of Technical and Non-Technical Skills

The evidence suggests a high level of consistency regarding the centrality of non-technical skills (NTS) for safe perioperative practice. The Delphi study, classified as high methodological quality, established robust consensus around five critical domains: communication, teamwork, leadership, situational awareness, and decision-making [18]. These findings demonstrate strong thematic consistency with subsequent qualitative studies [14,22,23] which describe perceived gains in clinical autonomy, prioritization, and stress management when supervision is structured and reflective.

However, most of these studies are based on self-reported data, which limits causal inference and the objective measurement of clinical impact. The empirical robustness of this domain is therefore conceptually consistent but methodologically moderate.

The quasi-experimental study [15] represents the only investigation with objective performance assessment, demonstrating a large effect size: a 47% reduction in simulated operative time, a 47% decrease in errors, and an overall performance improvement from 11.3% to 83.5% following the intervention. Although it focuses on immersive virtual reality training rather than exclusively on traditional clinical supervision, the findings suggest that structured learning approaches, integrated with supervisory processes, can produce substantial measurable gains. Nevertheless, the small sample size limits external generalizability.

Overall, the evidence in this domain shows strong conceptual convergence but moderate strength, due to the predominance of non-experimental designs and the limited use of objective safety indicators.

### 3.2. Structured Supervision Instruments

Two psychometric validation studies [18,20] provide methodologically robust evidence on structured instruments for the assessment of non-technical skills (NTS), namely NANTS-no and SPLINTS-no. High inter-rater reliability indices were reported (0.83–0.91), supporting both formative and summative use, thereby reinforcing evaluative consistency and the objectivity of the supervisory process. It was demonstrated that the systematic use of SPLINTS-no improves the quality of feedback, reduces subjectivity, and promotes alignment among supervisors [19]. Detailed descriptions of the NANTS-no and SPLINTS-no instruments are provided in the Table A3.

The convergence of these studies supports moderate to moderate–high strength of evidence regarding the psychometric quality and practical applicability of these instruments. However, a critical gap remains: there are no studies establishing a direct association between the use of these tools and the effective reduction in adverse events or measurable improvements in patient safety indicators.

### 3.3. Organizational Factors and Supervisor Qualification

The influence of the organizational context emerges as a cross-cutting determinant of the effectiveness of clinical supervision. The reduced time availability for supervision in the perioperative setting compared to other clinical environments suggests a tension between care demands and pedagogical functions [12]. This finding aligns with previous literature, which identifies productivity pressure and the lack of protected time as ongoing structural barriers [22]. Additionally, an association was demonstrated between high role ambiguity (77.5%) and failure to meet training objectives (55.2%), suggesting a significant organizational impact on the quality of clinical learning [21]. Although the cross-sectional nature of the study precludes causal inference, the consistency between quantitative and qualitative evidence reinforces the plausibility of this contextual effect.

In this domain, the evidence is considered moderate, limited by the predominance of observational studies and the absence of longitudinal or experimental research capable of estimating the magnitude of organizational effects.

### 3.4. Complementary Pedagogical Strategies

Interprofessional simulation [14] and immersive virtual reality [15] emerge as complementary strategies to direct supervision, enabling prior training of technical and non-technical skills in a controlled environment. Literature describes the development of competencies transferable to acute clinical situations, including interprofessional communication and decision-making under pressure [14]. One demonstrates significant objective gains in technical performance [15].

Despite thematic coherence and the magnitude of effect observed in the quasi-experimental study, the evidence remains emergent, with moderate–low strength, due to small sample sizes and the lack of multicenter replication.

### 3.5. Assessment of the Overall Strength of Evidence

Considering the substantial methodological heterogeneity of the included studies, the predominance of qualitative, cross-sectional, and psychometric designs, and the absence of randomized controlled trials or meta-analytic synthesis, a formal application of the GRADE framework was not considered appropriate. Nevertheless, to ensure interpretative rigor, the overall strength of evidence was categorized based on methodological quality assessed through JBI tools, consistency of findings across studies, hierarchy of evidence, and the presence of objective, measurable outcomes.

According to the hierarchy, the overall body of evidence predominantly falls within levels III and IV, corresponding mainly to cross-sectional observational studies, exploratory qualitative research, psychometric validation studies, and one quasi-experimental study without a control group [26]. No randomized controlled trials, multicenter longitudinal studies, or investigations with direct measurement of objective patient safety indicators (e.g., rates of adverse events or surgical complications) were identified.

In the domain of technical and non-technical skill development, the evidence can be classified as moderate in strength. There is high conceptual convergence across independent studies regarding the importance of communication, leadership, situational awareness, and decision-making. However, the predominance of self-reported data and the scarcity of objective clinical outcomes limit causal inference.

Regarding structured supervision instruments (NANTS-no and SPLINTS-no), the evidence indicates moderate to moderate–high strength in terms of psychometric properties, inter-rater reliability, and formative applicability. Nevertheless, there remains insufficient evidence establishing a direct association between the use of these instruments and a measurable reduction in adverse events.

With respect to organizational factors and supervisor qualification, the evidence is considered moderate, supported by thematic consistency across qualitative studies and statistical associations in cross-sectional research. However, the absence of longitudinal designs or controlled organizational interventions prevents estimation of the magnitude of contextual effects.

Overall, the synthesis allows the body of evidence to be classified as having moderate strength, with high conceptual consistency but structural methodological limitations that restrict external generalizability and the quantification of clinical impact. Thus, although there is strong plausibility of benefit associated with structured clinical supervision in the perioperative context, experimental and longitudinal studies are required to establish the magnitude of its effect on objective patient safety indicators.

## 4. Discussion

The findings of this systematic review highlight structured clinical supervision as a multifaceted process integrating education, reflective practice, professional development, and perioperative patient safety. Within highly complex surgical environments, supervision emerges not merely as an educational strategy but as an organizational and professional mechanism capable of supporting competency development, team integration, and safer clinical practice.

Despite the methodological and contextual heterogeneity of the included studies, a consistent pattern emerged regarding the relevance of structured supervision in strengthening both technical and non-technical competencies among perioperative nurses. Communication, leadership, situational awareness, decision-making, and reflective practice were repeatedly identified as essential competencies for safe perioperative care and appeared to be more effectively developed when supervision was formalized, deliberate, and supported by structured assessment and feedback processes. Instruments such as NANTS-no and SPLINTS-no illustrate the growing emphasis on standardized approaches to the assessment and development of non-technical skills within perioperative supervision.

These findings can also be interpreted through Patricia Benner’s Novice to Expert model, which conceptualizes professional competence as a progressive developmental process grounded in experiential learning and situated clinical practice. In perioperative environments characterized by rapid decision-making, technological complexity, and high-risk situations, structured clinical supervision appears to facilitate the transition from novice and advanced beginner stages toward higher levels of clinical proficiency by promoting reflective learning, supervised experiential exposure, and progressive professional autonomy.

Similarly, the findings may also be understood through the perspective of Situated Learning Theory, which emphasizes learning as a socially constructed process occurring through active participation in authentic clinical environments. From this perspective, supervision extends beyond technical instruction by supporting professional socialization, guided participation, interprofessional interaction, and the integration of less experienced nurses into perioperative teams and organizational cultures.

Importantly, the evidence synthesized in this review suggests that structured supervision may contribute to factors commonly associated with safer perioperative practice, including improved communication, supervisory consistency, teamwork, and professional confidence. In this context, internationally recognized safety strategies such as the World Health Organization Surgical Safety Checklist reinforce the importance of non-technical skills and standardized communication processes within surgical care. Clinical supervision may therefore function as a complementary mechanism supporting adherence to safety practices, reflective learning, and the strengthening of perioperative safety culture.

However, the relationship between clinical supervision and patient safety outcomes should be interpreted cautiously. Most included studies relied predominantly on self-reported perceptions, subjective experiences, and perceived competence rather than objective clinical indicators. Furthermore, none of the studies directly measured outcomes such as adverse events, surgical complications, healthcare-associated infections, reoperations, or mortality rates. Consequently, although supervision appears theoretically and organizationally linked to safer perioperative practice, direct causal evidence regarding its impact on measurable patient safety outcomes remains limited.

From a practical perspective, these findings reinforce the importance of developing formal organizational models for perioperative supervision, including structured supervisor preparation programs, protected supervisory time, competency-based assessment frameworks, and institutional policies supporting reflective practice and continuous professional development. The evidence also suggests that effective supervision should be recognized as a specialized professional competency requiring organizational support, pedagogical preparation, and integration into broader perioperative quality and safety strategies.

Taken together, these findings support the interpretation of structured clinical supervision as a central component of contemporary perioperative nursing practice, capable of integrating professional learning, competency development, reflective practice, and organizational support within highly demanding surgical environments.

The reviewed literature also highlights the importance of supervisor qualification and the institutional conditions that support supervised practice [12,13,19,21,24]. Studies on perioperative supervision indicate that specific training for supervisors, formal recognition of the role, and the provision of protected time are decisive factors for the effectiveness of the supervisory process [12,13]. These findings are consistent with evidence on role ambiguity and the challenges faced by students and professionals in training, demonstrating that environments with weak supervisory structures compromise learning, increase insecurity, and hinder the achievement of clinical objectives [19,21,22,23]. The consistency of these findings, obtained across different contexts and organizational settings, reinforces that clinical supervision depends not only on the individual actions of the supervisor but also on the existence of an institutional environment that values and sustains this practice. These findings are also consistent with broader theoretical and empirical literature on clinical supervision in nursing. Clinical supervision is conceptualized as an integrative process encompassing formative, restorative, and normative functions, supporting both professional development and accountability in clinical practice [9].

Studies examining the integration of students and professionals in adverse situations, such as during the COVID-19 pandemic, add a relevant perspective by demonstrating that consistent supervision provides both emotional and clinical support in high-pressure scenarios [19,22,23]. Similarly, studies exploring innovative approaches, such as immersive virtual reality and interprofessional simulation, show that these methodologies complement direct supervision by anticipating complex situations, optimizing supervised practice time, and contributing to the consolidation of competencies essential to safety [14,15]. Importantly, the findings of this review support the interpretation that simulation, immersive virtual reality, and structured assessment instruments should not be conceptualized as clinical supervision themselves but rather as educational and evaluative resources that may enhance supervisory effectiveness when embedded within formal supervisory frameworks. In this context of pedagogical innovation, structured clinical supervision emerges as a fundamental educational framework within perioperative practice. Clinical supervision is described as a cyclical process centered on observation, analysis, feedback, and continuous professional development rather than fault identification alone [27]. This perspective reinforces supervision as an ongoing learning pathway that supports reflective practice and professional growth. Furthermore, supervision functions are seen as a bridge between quality policies and professional practice, requiring collaborative dialogue and supportive leadership to strengthen learning environments [28]. Similarly, supervisory practices should contribute to continuous quality improvement, professional accountability, and practitioner autonomy [29]. These perspectives reinforce the interpretation that structured supervision in perioperative settings may contribute not only to professional competence development but also to safer and higher-quality clinical practice.

Simulation-based methodologies demonstrated effectiveness in the development of technical and non-technical competencies, while studies addressing continuing education revealed persistent gaps in training opportunities and professional support. This apparent contradiction suggests that the effectiveness of educational and supervisory interventions depends not only on the quality of the methodologies employed but also on their systematic, equitable, and organizationally supported implementation.

Similar divergences were identified regarding perceptions of the supervisor’s role. In some contexts, supervisors reported feeling valued, motivated, and professionally supported [12,13], whereas in other settings they described work overload, insufficient institutional recognition, and limited protected time for supervisory activities [13,21]. These tensions suggest that despite the widespread recognition of the importance of clinical supervision, its operationalization remains strongly influenced by institutional and organizational conditions. These findings are further supported by broader literature emphasizing supervision as a continuous, reflective, and developmental process.

Clinical supervision is conceptualized as a cyclical model grounded in observation, analysis, feedback, and reflective dialogue, promoting continuous professional growth and improvement in practice [27]. Supervision functions as a bridge between institutional quality standards and professional practice, reinforcing the importance of collaborative leadership, reflective dialogue, and organizational support [28]. Supervision is emphasized as a process that contributes to continuous quality improvement, professional accountability, and practitioner autonomy [29]. Collectively, these perspectives reinforce the interpretation that structured clinical supervision may contribute to safer perioperative environments, strengthened professional competence, and improved quality of care. From both theoretical and practical perspectives, the findings of this review demonstrate that structured clinical supervision may represent a relevant contribution to the quality and safety of surgical care by facilitating reflective learning, promoting objective feedback, and consolidating competencies essential to perioperative practice [19,20,22].

The integration of standardized assessment instruments, such as NANTS-no and SPLINTS-no, emerges as a central component in ensuring consistency, objectivity, and rigor within the supervisory process. The analyzed studies further indicate that investment in targeted supervisor training, the promotion of psychologically safe learning environments, and the incorporation of innovative educational technologies are key strategies for strengthening professional practice and fostering a culture of safety [12,13,19,21,22].

These findings are reinforced by broader literature highlighting the contribution of clinical supervision to healthcare quality and patient safety. Effective clinical supervision promotes reflective practice, professional support, improved team performance, and safer clinical environments [30]. Furthermore, the authors emphasize that structured supervisory processes may positively influence communication, professional development, and the overall effectiveness of care delivery. Collectively, these perspectives strengthen the interpretation that structured clinical supervision in perioperative settings may contribute to enhanced professional competence, stronger safety cultures, and improved patient outcomes.

Overall, the structured synthesis of the included studies enabled a critical comparison across heterogeneous methodological designs, supporting the identification of recurrent patterns, organizational determinants, and persistent limitations within the current evidence base.

Nevertheless, this review has important limitations that should be acknowledged. The predominance of observational, qualitative, and quasi-experimental studies limits the possibility of establishing direct causal relationships between structured clinical supervision and measurable clinical outcomes. Additionally, the heterogeneity of the included studies, the diversity of perioperative contexts addressed (including anesthesia, instrumentation, and general perioperative care), and the variability of methodological approaches restricted direct comparison and integration of findings across studies.

Another important limitation relates to the absence of standardized and objective patient safety indicators in most included studies. Although several studies reported improvements in communication, non-technical skills, reflective practice, professional confidence, and supervisory consistency—factors commonly associated with safer perioperative practice—none directly measured outcomes such as adverse events, surgical complications, incident rates, or mortality indicators. Therefore, the relationship between structured clinical supervision and patient safety should be interpreted cautiously and understood primarily as an indirect or theoretically supported association rather than a directly demonstrated causal effect. This limitation reinforces the need for future research incorporating robust and objective outcome measures capable of more accurately evaluating the impact of supervisory practices on perioperative safety performance.

It is also acknowledged that the temporal restriction adopted in this review, together with the inclusion of only studies with full-text availability, may have resulted in the exclusion of potentially relevant evidence. Although this criterion may have introduced selection bias, full-text access was considered essential to ensure comprehensive methodological appraisal, rigorous data extraction, and consistency in the synthesis and interpretation of evidence.

Despite these limitations, the findings of this review provide an important and conceptually structured contribution to the understanding of clinical supervision within perioperative nursing. The evidence synthesized reinforces the relevance of structured supervision as an organizational, educational, and reflective process capable of supporting professional development, strengthening non-technical competencies, and contributing to safer perioperative practice.

From an organizational perspective, the findings highlight the need for healthcare institutions to recognize clinical supervision as a strategic component of perioperative nursing practice by ensuring adequate supervisory structures, protected training opportunities, institutional support, and continuous professional development for both supervisors and supervisees. Furthermore, the review identifies priority areas for future research, including the development of perioperative-specific supervisor training models, longitudinal studies examining the influence of supervision on professional retention and career progression, and mixed-methods investigations capable of linking supervisory practices with objective patient safety and quality-of-care outcomes.

In summary, the findings of this review suggest that structured clinical supervision represents an important component of professional development within perioperative settings. The integration of supervision with continuing education, reflective practice, structured assessment frameworks, and complementary educational technologies emerges as a promising strategy for strengthening perioperative nursing teams and supporting improvements in the quality and safety of surgical care.

## 5. Conclusions

The findings of this review suggest that structured clinical supervision may contribute to safer perioperative practice by strengthening professional competencies, reflective practice, communication, and supervisory consistency. However, further research incorporating objective clinical safety indicators is required to clarify the direct impact of supervision on patient safety outcomes within perioperative settings.

This systematic review synthesized contemporary scientific evidence regarding the role and impact of structured clinical supervision in perioperative nursing practice. The analysis shows that supervision constitutes an important organizational, educational, and reflective component of professional development within highly complex surgical environments.

The included studies consistently suggest that formalized models of clinical supervision support the development of both technical and non-technical competencies among perioperative nurses. Improvements were identified in professional confidence, clinical decision-making, interprofessional communication, reflective practice, and the integration of students and newly qualified professionals into operating room settings. Collectively, these findings reinforce the potential of structured supervision to support safer, more consistent, and professionally supportive perioperative practice.

The review also enabled the identification of factors that facilitate or hinder effective supervisory processes. The evidence indicates that supervision supported by trained supervisors, structured assessment frameworks, and standardized instruments contributes to the creation of safe learning environments and strengthens supervisory consistency. Instruments such as NANTS-no and SPLINTS-no appear particularly relevant for promoting objective observation and structured feedback within perioperative teams. Conversely, role ambiguity, insufficient institutional support, limited protected teaching time, and unclear supervisory responsibilities were consistently identified as barriers compromising the effectiveness of supervision and the achievement of educational objectives.

From a theoretical perspective, the findings reinforce clinical supervision as a structuring element of contemporary perioperative nursing practice by integrating professional development, competency assessment, reflective learning, and organizational support. At a practical level, the evidence suggests that investment in formal supervisory models, supervisor preparation, validated assessment tools, and structured educational strategies may strengthen the quality and consistency of perioperative nursing practice.

Additionally, complementary pedagogical methodologies such as interprofessional simulation and immersive virtual reality demonstrated potential to support supervisory processes by facilitating the prior development of complex competencies and optimizing learning opportunities within clinical environments. Importantly, these approaches should not be conceptualized as clinical supervision itself but rather as complementary educational resources capable of strengthening supervisory effectiveness when integrated into formal supervisory frameworks.

From an organizational and policy perspective, the findings highlight the importance of recognizing clinical supervision as a strategic component of perioperative nursing practice. Ensuring adequate institutional support, continuous supervisor training, protected educational time, and appropriate supervisory structures may contribute to the development of more consistent supervisory practices and improved professional support within operating room teams.

In summary, this systematic review provides a conceptually structured synthesis of the current evidence regarding structured clinical supervision in perioperative nursing. Although direct causal relationships between supervision and objective patient safety outcomes remain insufficiently demonstrated, the evidence suggests that structured supervision may support factors associated with safer perioperative practice, professional development, and continuous improvement in the quality of surgical care. The findings also reinforce the need for future research using robust methodological designs and objective clinical outcome indicators to further clarify the impact of supervision in perioperative settings.

## Figures and Tables

**Table 1:** Methodological Quality Assessment of Included Studies (JBI).

Study	Design	JBI Tool Applied	Criteria Met (n/N)	Percentage	Classification
Sirevåg, Tjoflåt, et al. (2021) [18]	Modified Delphi	Analytical Cross-Sectional	5/6	83%	High
Sirevåg, Aamodt, et al. (2021) [19]	Psychometric validation	Analytical Cross-Sectional	6/8	75%	Moderate–High
Flynn et al. (2021) [20]	Psychometric validation	Analytical Cross-Sectional	6/8	75%	Moderate–High
Harper et al. (2023) [12]	Secondary analysis	Analytical Cross-Sectional	6/8	75%	Moderate–High
Abdelaziz et al. (2025) [21]	Correlational	Analytical Cross-Sectional	6/8	75%	Moderate–High
Edwards et al. (2021) [15]	Quasi-experimental (pre–post)	Quasi-Experimental	7/9	78%	Moderate–High
Hedlund et al. (2024) [22]	Qualitative	Qualitative Research	7/10	70%	Moderate
Jaensson et al. (2025) [23]	Qualitative	Qualitative Research	7/10	70%	Moderate
Kaldheim et al. (2023) [14]	Qualitative	Qualitative Research	7/10	70%	Moderate
Shin & Kim (2021) [24]	Cross-sectional	Analytical Cross-Sectional	4/8	50%	Moderate–Low
Ssemakula (2025) [25]	Cross-sectional	Analytical Cross-Sectional	3/8	38%	Low
Wakefield (2021) [13]	Professional report	Text and Opinion	6/6	100% *	High (Text and Opinion)

* High quality within the Text and Opinion category (non-empirical evidence).

**Table 2:** Characteristics and Main Findings of Included Studies.

Authors Year	Country	Title	Objective	Methodology	Sample	Results
Sirevåg, I., Tjoflåt, I., & Hansen, B.S. (2021) [18]	Norway	A Delphi study identifying operating room nurses’ non-technical skills	To identify the non-technical skills of operating room nurses (scrub and circulating nurses).	Modified Delphi consensus study conducted in three online rounds using descriptive quantitative and deductive thematic analysis. No psychometric evaluation was performed.	106 experienced perioperative nurses.	Consensus was reached on five core non-technical skill domains: communication, teamwork, leadership, situational awareness, and decision-making. Additional competencies included independent decision-making, diversified leadership, and critical awareness relevant to perioperative nursing practice.
Ssemakula, T.N. (2025) [25]	United Kingdom	Do the current continuous professional development approaches for registered nurses and operating department practitioners within perioperative care meet their learning needs?	To evaluate the effectiveness of current continuing professional development (CPD) approaches in meeting the learning needs of registered nurses (RNs) and operating department practitioners (ODPs) in perioperative care within an NHS Trust hospital in the United Kingdom.	Descriptive cross-sectional study using quantitative descriptive and thematic qualitative analysis.	76 participants.	Four themes emerged: knowledge acquisition, patient-centered care, tailored learning, and organizational support. Participants emphasized the importance of CPD, while barriers included inconsistent feedback, staff shortages, and time constraints.
Jaensson, M., Hedlund, J., & Blomberg, K. (2025) [23]	Sweden	Experience of Student Nurse Anaesthetists Learning in the Operating Room During the COVID-19 Pandemic: A Qualitative Interview Study.	To explore the learning of student nurse anesthetists (SNAs) in the operating room during the COVID-19 pandemic.	Exploratory qualitative study using semi-structured interviews and thematic analysis according to Braun and Clarke.	21 participants, including former student nurse anesthetists and clinical supervisors from six Swedish regions.	Clinical learning remained central despite the COVID-19 pandemic. Main challenges included unstable learning environments, limited supervision continuity, stress, fear of infection, and reduced learning opportunities. Students and supervisors demonstrated resilience in sustaining active learning.
Edwards, T.C., Patel, A., Szyszka, B., Coombs, A.W., Liddle, A.D., Kucheria, R., Cobb, J.P., & Logishetty, K. (2021) [15]	United Kingdom	Immersive virtual reality enables technical skill acquisition for scrub nurses in complex revision total knee arthroplasty	To investigate the impact of an immersive virtual reality (iVR) curriculum on the acquisition of technical skills by scrub nurses for the selection, assembly, and handling of instruments in complex revision total knee arthroplasty surgery.	Quasi-experimental pre–post study using immersive virtual reality (iVR) training with simulated and real-world assessments.	10 scrub nurses completed the study.	Immersive virtual reality training significantly reduced operative time and technical errors while improving confidence, performance, and anxiety management during complex surgical procedures.
Hedlund, J., Blomberg, K., Hjelmqvist, H., & Jaensson, M. (2024) [22]	Sweden	Nurse anaesthetists’ experiences of student nurse anaesthetist learning during clinical practice: a qualitative interview study	To describe nurse anesthetist supervisors’ experiences of student nurse anesthetists’ learning during clinical practice in the operating room.	Qualitative study using semi-structured interviews and thematic analysis.	12 nurse anesthetist supervisors from 6 counties in Sweden (urban and rural hospitals).	Clinical supervision supported reflective learning, student adaptation, assessment processes, and transition to perioperative practice despite workload pressures and limited supervision time.
Shin, Y.Y. & Kim, S.S. (2021) [24]	South Korea	Operating Room Nurses Want Differentiated Education for Perioperative Competencies—Based on the Clinical Ladder	To identify perioperative competencies and educational needs for competency improvement among operating room (OR) nurses according to the clinical ladder.	Descriptive cross-sectional study.	318 operating room nurses from tertiary hospitals in Seoul and metropolitan areas.	Operating room nurses emphasized the need for differentiated perioperative education aligned with clinical competency progression. Collaboration was the highest-rated competency domain, whereas leadership scored lowest.
Wakefield, E. (2021) [13]	Australia	Preceptoring the preceptors: Empowering and sustaining our profession	To describe the implementation of an educational program to support, empower, and sustain perioperative supervisors in a semi-rural operating room, enhancing their effectiveness in teaching, supervision, and clinical assessment.	Experience report describing an educational support program for perioperative supervisors.	Group of experienced perioperative supervisors (number not specified) from a semi-rural operating room providing obstetric and general surgery services.	Supervisor training promoted reflective practice, feedback skills, teamwork, and alignment of learning objectives despite limited protected supervision time.
Kaldheim, H.K.A., Fossum, M., Munday, J., Creutzfeldt, J., & Slettebø, Å. (2023) [14]	Norway	Professional competence development through interprofessional simulation-based learning assists perioperative nurses in postgraduation acute clinical practice situations: A qualitative study	To explore newly graduated perioperative nurses’ experiences of interprofessional simulation-based learning (ISBL) during postgraduate education and to investigate whether and how this approach contributed to the development of their professional competence in managing acute clinical situations.	Exploratory qualitative study using semi-structured interviews and hermeneutic phenomenological analysis.	16 newly graduated perioperative nurses from 5 higher education institutions in Norway.	Interprofessional simulation enhanced teamwork, communication, reflection, decision-making, and professional identity development in acute perioperative situations. Debriefing was identified as essential for reflective learning and patient safety.
Flynn, F.M., Valeberg, B.T., Tønnessen, S., & Bing-Jonsson, P.C. (2020) [20]	Norway	Psychometric testing of a structured assessment instrument for non-technical skills NANTS-no -(Nurse Anaesthetists’ Non-Technical Skills, Norwegian version),for use in clinical supervision of student nurse anaesthetists	To evaluate the psychometric properties of the structured behavioral assessment instrument NANTS-no (Nurse Anaesthetists’ Non-Technical Skills—Norway); to estimate whether reliable assessments of non-technical skills (NTS) can be conducted following participation in a workshop; and to assess the acceptability and usability of the instrument.	Exploratory psychometric validation study.	46 nurse anesthetists from 4 hospital trusts in Norway.	NANTS-no demonstrated acceptable inter-rater reliability and was considered useful for feedback, reflection, skill development, and structured student assessment in clinical supervision.
Abdelaziz, M.M.A., Saleh, M.S.M., Aysha, Z.M., & Abou Shaheen, R.A. (2025) [21]	Egypt	Role ambiguity and nursing interns’ achievement of clinical rotation goals: a correlational study	To explore the influence of role ambiguity on the achievement of clinical rotation objectives among nursing interns.	Descriptive cross-sectional correlational study.	900 nursing interns from Tanta University Hospitals (Emergency Hospital and Main University Hospitals).	High role ambiguity and insufficient supervision were associated with difficulties in achieving clinical learning objectives and reduced training satisfaction. Main deficits included limited orientation, inconsistent feedback, and inadequate psychosocial support.
Sirevåg, I., Aamodt, K.H., Mykkeltveit, I., & Bentsen, S.B. (2021) [19]	Norway	Student supervision using the Scrub Practitioners’ List of Intraoperative Non-Technical Skills (SPLINTS-no): A qualitative study	To explore operating room nurse supervisors’ experiences of using the SPLINTS-no instrument (Norwegian version) in supervising the non-technical skills of operating room nursing students.	Exploratory qualitative study using inductive qualitative content analysis.	10 operating room nurse supervisors from a Norwegian university hospital.	SPLINTS-no improved feedback quality, supervisory consistency, reflection, and communication regarding non-technical skills within perioperative teams.
Harper, M.G., Whiteside, D., Warren, J.I., MacDonald, R., & Ulrich, B. (2023) [12]	United States of America	The Association for Nursing Professional Development National Preceptor Practice Analysis Study: An Exploration of Precepting in Perioperative Settings	To analyze the responses of perioperative supervisors from the 2020 Preceptor Practice Analysis Study, to compare perceptions of perioperative versus non-perioperative supervisors, and to assess whether perceptions differ with or without training.	Secondary analysis of the 2020 National Preceptor Practice Analysis Study.	400 perioperative nurse supervisors.	Perioperative supervisors valued all preceptor roles but reported reduced supervision time and organizational constraints within perioperative settings.

## Data Availability

All data are provided via tables in the text and via text and tables in Appendix A. The included articles are available via the electronic databases utilized in this study.

## Public Involvement Statement

No patients or members of the public were involved in the design, conduct, reporting, or dissemination plans of this systematic review.

## Guidelines and Standards Statement

This systematic review was conducted and reported in accordance with the Preferred Reporting Items for Systematic Reviews and Meta-Analyses (PRISMA 2020) guidelines. The review protocol was prospectively registered in the International Prospective Register of Systematic Reviews (PROSPERO).

## Use of Artificial Intelligence

The CONSENSUS platform was used as an artificial intelligence-assisted tool to support the literature search process. Its use was limited to identifying potentially relevant scientific publications. All study screening, eligibility assessment, data extraction, critical appraisal, synthesis of findings, interpretation of results, and manuscript writing were performed by the authors. The authors reviewed and verified all retrieved information and take full responsibility for the content of this manuscript.

## Appendix A

### Appendix A.1. Search Strategies

**Table A1:** Database-specific search strategies, limits, dates of execution, and number of records retrieved.

Database/Platform	Search Date	Search String	Limits/Filters	Records Retrieved
PubMed	17 October 2025	(Nurs* OR “Perioperative Nursing” OR “Operating Room Nursing”) AND (“operating room” OR “operating theatre” OR surgery) AND (preceptorship OR “clinical supervision”) AND (“Continuing Nursing Education” OR “Nursing Education” OR “Quality of Health Care”)	Publication date: 2020–2025; Languages: English, Portuguese, Spanish; Full text available	101
Web of Science	17 October 2025	(Nurs* OR “Perioperative Nursing” OR “Operating Room Nursing”) AND (“operating room” OR “operating theatre” OR surgery) AND (preceptorship OR “clinical supervision”) AND (“Continuing Nursing Education” OR “Nursing Education” OR “Quality of Health Care”)	Publication date: 2020–2025; Languages: English, Portuguese, Spanish	14
EBSCOhost (CINAHL, MEDLINE Complete)	17 October 2025	(Nurs* OR “Perioperative Nursing” OR “Operating Room Nursing”) AND (“operating room” OR “operating theatre” OR surgery) AND (preceptorship OR “clinical supervision”) AND (“Continuing Nursing Education” OR “Nursing Education” OR “Quality of Health Care”)	Publication date: 2020–2025; Languages: English, Portuguese, Spanish; Full text available	2
SciELO	17 October 2025	(Nurs* OR “Perioperative Nursing” OR “Operating Room Nursing”) AND (“operating room” OR “operating theatre” OR surgery) AND (preceptorship OR “clinical supervision”) AND (“Continuing Nursing Education” OR “Nursing Education” OR “Quality of Health Care”)	Publication date: 2020–2025; Languages: English, Portuguese, Spanish	0
BVS (Biblioteca Virtual em Saúde)	17 October 2025	(Nurs* OR “Perioperative Nursing” OR “Operating Room Nursing”) AND (“operating room” OR “operating theatre” OR surgery) AND (preceptorship OR “clinical supervision”) AND (“Continuing Nursing Education” OR “Nursing Education” OR “Quality of Health Care”)	Publication date: 2020–2025; Languages: English, Portuguese, Spanish	0
CONSENSUS	17 October 2025	(Nurs* OR “Perioperative Nursing” OR “Operating Room Nursing”) AND (“operating room” OR “operating theatre” OR surgery) AND (preceptorship OR “clinical supervision”) AND (“Continuing Nursing Education” OR “Nursing Education” OR “Quality of Health Care”)	Publication date: 2020–2025; Languages: English, Portuguese, Spanish	11

**Table A2:** PRISMA 2020 checklist for reporting of the systematic review.

Section	Item	Description	Reported	Location
Title	1	Identify as systematic review	Yes	Title
Abstract	2	Structured summary	Yes	Abstract
Introduction	3	Rationale	Yes	Introduction
Introduction	4	Objectives	Yes	End of Introduction
Methods	5	Protocol and registration	Yes	PROSPERO
Methods	6	Eligibility criteria	Yes	Section 2.2
Methods	7	Information sources	Yes	Section 2.1
Methods	8	Search strategy	Yes	Section 2.1 + Appendix A
Methods	9	Selection process	Yes	Section 2.3
Methods	10	Data collection	Yes	Section 2.5
Methods	11	Data items	Yes	Section 2.5
Methods	12	Risk of bias	Yes	Section 2.4
Methods	13	Effect measures	Partial	No meta-analysis
Methods	14	Synthesis methods	Yes	Section 2.5
Methods	15	Heterogeneity	Partial	Narrative
Methods	16	Additional analyses	No	Not done
Results	17	Study selection	Yes	Figure 1
Results	18	Study characteristics	Yes	Table 2
Results	19	Risk of bias	Yes	Table 1
Results	20	Individual results	Yes	Table 2
Results	21	Synthesis results	Yes	Section 3
Results	22	Heterogeneity	Partial	Narrative
Results	23	Additional analyses	No	Not done
Discussion	24	Summary of evidence	Yes	Discussion
Discussion	25	Limitations	Yes	Discussion
Discussion	26	Implications	Yes	Discussion
Other	27	Funding	Yes	Funding
Other	28	Conflicts of interest	Yes	COI
Other	29	Data availability	Yes	Statement

**Table A3:** Overview of the NANTS-no and SPLINTS-no instruments used in perioperative clinical supervision.

Instrument	Full Name	Purpose	Main Domains/Components	Application in Clinical Supervision	Psychometric Properties/Key Findings	Reference
NANTS-no	Nurse Anaesthetists’ Non-Technical Skills—Norway	Structured assessment instrument designed to evaluate non-technical skills among student nurse anesthetists and support formative and summative assessment processes.	Situational awareness; Decision-making; Communication; Teamwork	Used during clinical supervision and assessment of student nurse anesthetists in perioperative and anesthesia settings. Supports structured feedback, reflective practice, and standardized evaluation of non-technical competencies	Flynn et al. (2021) [20] demonstrated acceptable inter-rater reliability with one assessor (0.83) and high reliability with two assessors (0.91). Participants considered the instrument useful for skill development, feedback, and critical reflection.	Flynn, F.M., Valeberg, B.T., Tønnessen, S., & Bing-Jonsson, P.C. (2021) [20]. Psychometric testing of a structured assessment instrument for non-technical skills (NANTS-no) for use in clinical supervision of student nurse anesthetists.
SPLINTS-no	Scrub Practitioners’ List of Intraoperative Non-Technical Skills—Norway	Structured instrument developed to support the assessment and supervision of non-technical skills among operating room nursing students and scrub practitioners.	Situational awareness; Communication and teamwork; Task management	Applied during perioperative clinical supervision to facilitate objective assessment and improve the quality and consistency of supervisory feedback. Promotes shared terminology and structured reflection on non-technical skills.	Sirevåg, Aamodt, et al. (2021) [19] reported that the instrument improved supervisory consistency, reduced vague feedback, enhanced reflection, and strengthened supervisors’ confidence in student assessment.	Sirevåg, I., Aamodt, K.H., Mykkeltveit, I., & Bentsen, S.B. (2021) [19]. Student supervision using the Scrub Practitioners’ List of Intraoperative Non-Technical Skills (SPLINTS-no): A qualitative study.

### Appendix A.2. Supplementary Methodological Information

The instruments NANTS-no (Nurse Anaesthetists’ Non-Technical Skills—Norway) and SPLINTS-no (Scrub Practitioners’ List of Intraoperative Non-Technical Skills—Norway) are structured assessment tools specifically developed to support the evaluation and supervision of non-technical skills in perioperative nursing practice.

Both instruments are grounded in patient safety principles and emphasize the relevance of non-technical competencies, including communication, teamwork, situational awareness, leadership, task management, and decision-making within high-complexity surgical environments.

NANTS-no was designed for use in the supervision and assessment of student nurse anesthetists. The instrument provides a structured framework that facilitates objective observation, formative feedback, reflective practice, and consistency among supervisors. Evidence from psychometric testing demonstrated satisfactory inter-rater reliability and high acceptability among clinical supervisors, supporting its applicability in both formative and summative assessment processes.

SPLINTS-no was adapted for the perioperative nursing context to support the supervision of operating room nursing students and scrub practitioners. The instrument contributes to the standardization of supervisory practices by providing a common professional language for describing and evaluating non-technical skills. Studies included in this review indicate that SPLINTS-no improves the quality of feedback, promotes reflective learning, and enhances consistency in clinical supervision.

The integration of structured instruments such as NANTS-no and SPLINTS-no into perioperative clinical supervision may contribute to more rigorous assessment processes, improved supervisory communication, and greater emphasis on patient safety culture within surgical settings.

## References

[1] Gomes J.A., Martins M.M., Tronchin D., Fernandes C.S. (2020). Perceção dos enfermeiros sobre a qualidade em saúde no bloco operatório. Rev. Enferm. Ref..

[2] Rebelo S.M.d.S.R. (2013). Segurança do Doente no Bloco Operatório. Master’s Thesis.

[3] Martinez-Nicolas I., Arnal-Velasco D., Romero-García E., Fabregas N., Otero Y.S., Leon I., Bartakke A.A., Silva-Garcia J., Rodriguez A., Valli C. (2024). Perioperative patient safety recommendations: Systematic review of clinical practice guidelines. BJS Open.

[4] Danski M.T.R., da Silva C.M., Cunha M.G.d.B. (2023). Assistência perioperatória de enfermagem voltada à segurança do paciente cirúrgico: Uma revisão integrativa. Rev. SOBECC.

[5] Bradley P., King R., Bev L., Marks P., Murphy S., Sharrock J. (2019). Position Statement Clinical Supervision for Nurses & Midwives.

[6] Kim N.Y., Jeong S.Y. (2021). Perioperative patient safety management activities: A modified theory of planned behavior. PLoS ONE.

[7] Rodrigues M., Carpinteiro D., Deus I., Bengalinha P., Duro R., Silva C., Mendonça S. (2025). Supervisão Clínica em Estudantes de Enfermagem na Promoção da Segurança do Paciente: Scoping Review. Rev. Ibero-Am. De Saúde E Envelhec..

[8] Singh B.C., Arulappan J. (2023). Operating Room Nurses’ Understanding of Their Roles and Responsibilities for Patient Care and Safety Measures in Intraoperative Practice. SAGE Open Nurs..

[9] Proctor B. (2008). Group Supervision: A Guide to Creative Practice.

[10] Cutcliffe J.R., Sloan G., Bashaw M. (2018). A systematic review of clinical supervision evaluation studies in nursing. Int. J. Ment. Health Nurs..

[11] Bifarin O., Stonehouse D. (2017). Clinical supervision: An important part of every nurse’s practice. Br. J. Nurs..

[12] Harper M.G., Whiteside D., Warren J.I., MacDonald R., Ulrich B. (2023). The Association for Nursing Professional Development National Preceptor Practice Analysis Study: An Exploration of Precepting in Perioperative Settings. AORN J..

[13] Wakefield E. (2021). Preceptoring the preceptors Empowering and sustaining our profession. Aust. Nurs. Midwifery J..

[14] Kaldheim H.K.A., Fossum M., Munday J., Creutzfeldt J., Slettebø Å. (2023). Professional competence development through interprofessional simulation-based learning assists perioperative nurses in postgraduation acute clinical practice situations: A qualitative study. J. Clin. Nurs..

[15] Edwards T.C., Patel A., Szyszka B., Coombs A.W., Liddle A.D., Kucheria R., Cobb J.P., Logishetty K. (2021). Immersive virtual reality enables technical skill acquisition for scrub nurses in complex revision total knee arthroplasty. Arch. Orthop. Trauma Surg..

[16] dos Santos Y.H.F., Bolina A.F., Bueno A.d.A., Paranaguá T.T.d.B., de Souza M.A., Aguiar S.C.d.S. (2024). Nursing supervision in hospital settings: From the entry into the role to the organizational planning process. Rev. Enferm..

[17] Bento A. (2012). Como fazer uma revisão da literatura: Considerações teóricas e práticas. Rev. JA (Assoc. Académica Da Univ. Da Madeira).

[18] Sirevåg I., Tjoflåt I., Hansen B.S. (2021). A Delphi study identifying operating room nurses’ non-technical skills. J. Adv. Nurs..

[19] Sirevåg I., Aamodt K.H., Mykkeltveit I., Bentsen S.B. (2021). Student supervision using the Scrub Practitioners’ List of Intraoperative Non-Technical Skills (SPLINTS-no): A qualitative study. Nurse Educ. Today.

[20] Flynn F.M., Valeberg B.T., Tønnessen S., Bing-Jonsson P.C. (2021). Psychometric Testing of a Structured Assessment Instrument for Non-technical Skills (NANTS-no) for Use in Clinical Supervision of Student Nurse Anesthetists. J. Nurs. Meas..

[21] Abdelaziz M.M.A., Saleh M.S.M., Aysha Z.M., Shaheen R.A.E.-M.A. (2025). Role ambiguity and nursing interns’ achievement of clinical rotation goals: A correlational study. BMC Nurs..

[22] Hedlund J., Blomberg K., Hjelmqvist H., Jaensson M. (2024). Nurse anaesthetists’ experiences of student nurse anaesthetist learning during clinical practice: A qualitative interview study. BMC Nurs..

[23] Jaensson M., Hedlund J., Blomberg K. (2025). Experience of Student Nurse Anesthetists’ Learning in the Operating Room During the COVID-19 Pandemic: A Qualitative Interview Study. J. Perianesthesia Nurs..

[24] Shin Y.Y., Kim S.S. (2021). Operating room nurses want differentiated education for perioperative competencies—Based on the clinical ladder. Int. J. Environ. Res. Public Health.

[25] Ssemakula T.N. (2025). Do the current continuous professional development approaches for registered nurses and operating department practitioners within perioperative care meet their learning needs?. J. Perioper. Pract..

[26] Melnyk B.M., Fineout-Overholt E. (2019). Evidence-Based Practice in Nursing & Healthcare: A Guide to Best Practice.

[27] Lahri M., El Ouanjli H. (2025). Clinical Supervision in Education: A Formative Framework for Enhancing Instruction and Professional Development. Int. J. Linguist. Lit. Transl..

[28] Purnawati, Hamidah, Apriliyani, Warman (2025). Transformation of Educational Supervision in Improving School Quality Between Classical Theory and Contemporary Practice. Int. J. Educ. Life Sci..

[29] Fatimah M., Hartanto R., Priyanto C. (2025). Konsep Supervisi Pendidikan. Tsaqofah.

[30] Snowdon D.A., Leggat S.G., Taylor N.F. (2017). Does clinical supervision of healthcare professionals improve effectiveness of care and patient experience? A systematic review. BMC Health Serv. Res..

